# Buccal Articaine Infiltration as a Minimally Invasive Technique for Mandibular Primary Molars: A Systematic Review

**DOI:** 10.3390/dj14100620

**Published:** 2026-09-26

**Authors:** Rasa Mladenovic, Marko Milosavljevic, Milica Jovanovic, Andjela Milojevic Samanovic, Nikola Prodanovic, Kristina Mladenovic, Milan Rabrenovic, Ervin Taso, Igor Grbic, Tijana Prodanovic

**Affiliations:** 1 Department of Dentistry, Faculty of Medical Sciences, University of Kragujevac, 34000 Kragujevac, Serbia; micamonro@gmail.com (M.J.); andjela-kg@hotmail.com (A.M.S.); 2 Dental Medicine Clinic Dentokids, 34000 Kragujevac, Serbia; 3 Department of Surgery, Faculty of Medical Science, University of Kragujevac, 34000 Kragujevac, Serbia; nikolaprodanovickg@gmail.com; 4 Clinic for Orthopedic and Trauma Surgery, University Clinical Center Kragujevac, 34000 Kragujevac, Serbia; 5 Department of Physical Medicine and Rehabilitation, University Clinical Center of Kragujevac, 34000 Kragujevac, Serbia; kristinamladenovic1990@gmail.com; 6 Faculty of Medical Sciences, University of Kragujevac, 34000 Kragujevac, Serbia; milan13rabrenovic@gmail.com; 7 Military Medical Academy, 11000 Belgrade, Serbia; 8 Clinic for Stomatology, Military Medical Academy, 11000 Belgrade, Serbia; drervintaso@gmail.com; 9 Department of Philosophical and Sports Sciences, State University of Novi Pazar, 36300 Novi Pazar, Serbia; gbigor86@gmail.com; 10 Department of Pediatrics, Faculty of Medical Sciences University of Kragujevac, University of Kragujevac, 34000 Kragujevac, Serbia; tijanaprodanovic86@gmail.com; 11 Center for Neonatology, Pediatric Clinic, University Clinical Center Kragujevac, 34000 Kragujevac, Serbia

**Keywords:** local anesthesia, articaine, buccal infiltration, minimally invasive dentistry

## Abstract

**Background/Objectives**: Effective pain control is essential for successful dental treatment in children. Although the inferior alveolar nerve block remains the standard technique for achieving mandibular anesthesia, it is associated with injection discomfort, prolonged soft tissue numbness, and an increased risk of postoperative self-inflicted soft tissue injury. Buccal infiltration with 4% articaine has emerged as a promising minimally invasive alternative because of its favorable diffusion properties. This systematic review aimed to evaluate the clinical efficacy of buccal infiltration with 4% articaine for local anesthesia of primary mandibular molars in pediatric patients. **Methods**: This systematic review was conducted according to the PRISMA 2020 guidelines. Electronic databases were searched to identify randomized controlled clinical trials evaluating buccal infiltration with 4% articaine in primary mandibular molars. Owing to methodological heterogeneity, the findings were synthesized qualitatively. **Results**: Five randomized controlled trials met the eligibility criteria. Buccal infiltration with 4% articaine demonstrated high anesthetic success and was generally associated with comparable pain scores, favorable patient comfort, and favorable postoperative outcomes compared with conventional anesthetic techniques. However, heterogeneity was observed in clinical procedures, comparator interventions, and outcome assessment methods. **Conclusions**: Current evidence suggests that buccal infiltration with 4% articaine is an effective and clinically comparable, minimally invasive anesthetic technique for selected procedures involving primary mandibular molars in children.

## 1. Introduction

Pain control is one of the most important determinants of successful dental treatment in pediatric patients. Pain is a complex sensory and emotional experience that may substantially influence a child’s behavior, emotional state, and ability to cooperate during dental procedures. Unlike adults, children may have a limited ability to anticipate, understand, communicate, and cope with painful stimuli, making effective pain prevention and management particularly important in this population. The perception of dental pain is also influenced by psychological and behavioral factors, including fear, anxiety, previous dental experiences, expectations, and the child’s level of cognitive and emotional development. Consequently, the experience of pain during dental treatment cannot be considered independently of the child’s overall psychological response to the dental environment [1].

Painful dental experiences, particularly when unexpected or inadequately controlled, may have lasting effects on a child’s perception of dental care. Negative pain-related experiences during dental procedures may contribute to the development or reinforcement of dental fear and anxiety, avoidance of future dental care, and reduced cooperation during subsequent visits. Moreover, anticipatory fear of pain may increase anxiety even before treatment begins and may result in behavioral resistance, making subsequent procedures more challenging for both the child and the dental team. This interaction may create a negative cycle in which anxiety increases the perception of pain, pain reduces cooperation, and difficult treatment experiences further reinforce dental fear and avoidance. Such experiences during childhood may therefore influence patterns of dental attendance and oral health-related behaviors later in life [2,3].

Effective pain management is consequently important not only for ensuring the child’s comfort during an individual procedure but also for facilitating appropriate clinical treatment and promoting positive long-term attitudes toward dental care. Adequate pain control can improve cooperation, reduce distress, facilitate communication between the child and the dental team, and contribute to a more predictable treatment experience. From a clinical perspective, the prevention of procedural pain should therefore be regarded as an integral component of pediatric dental care rather than merely an adjunct to treatment. Achieving profound, reliable, and appropriately timed local anesthesia is essential for minimizing procedural pain and supporting successful dental treatment in children [4].

The inferior alveolar nerve block (IANB) has traditionally been regarded as the standard technique for achieving mandibular anesthesia during procedures involving primary mandibular molars [5]. However, its use in children is associated with several limitations and potential complications. Administration of the nerve block itself may be painful and psychologically distressing for pediatric patients, while prolonged numbness of the lower lip, cheek, and tongue increases the risk of postoperative self-inflicted soft tissue injury.

Postoperative lip, cheek, and tongue biting is one of the most common complications associated with IANB in children [6]. These injuries may result in pain, swelling, ulceration, and increased anxiety for both children and their caregivers. Consequently, considerable interest has emerged in developing less invasive anesthetic techniques capable of providing adequate analgesia while minimizing postoperative adverse effects.

Articaine is an amide local anesthetic with a distinctive chemical structure characterized by the presence of a thiophene ring and an additional ester group. The thiophene ring contributes to the relatively high lipid solubility of the molecule, which may facilitate diffusion through soft and hard tissues [7]. In contrast to other commonly used amide local anesthetics, articaine contains an additional ester group that allows for substantial metabolism by plasma carboxylesterases, contributing to its rapid systemic hydrolysis. Compared with lidocaine, articaine demonstrates greater bone penetration, which may facilitate effective infiltration anesthesia even in the mandibular region.

The anatomical characteristics of the pediatric mandible may further contribute to the success of infiltration anesthesia. The cortical bone surrounding primary teeth in children is thinner, less dense, and more porous than that of the permanent dentition in adults. These anatomical features may facilitate diffusion of the anesthetic solution toward the apical region of primary teeth and may reduce the need for deeper nerve block techniques.

Several randomized controlled trials (RCTs) have evaluated the efficacy of buccal infiltration with articaine (BI) in primary mandibular molars [8,9]. However, differences in study design, clinical procedures, outcome measures, and comparator interventions have resulted in heterogeneous findings. Although systematic reviews addressing this topic were published in 2021, additional clinical evidence has since become available, and a more focused evaluation of buccal infiltration with articaine specifically for primary mandibular molars in children is warranted. Therefore, this systematic review aimed to provide an updated synthesis of the available evidence and to evaluate the clinical efficacy of buccal infiltration with 4% articaine during dental procedures involving primary mandibular molars in pediatric patients.

## 2. Materials and Methods

### 2.1. Search Strategy

This systematic review was conducted according to the Preferred Reporting Items for Systematic Reviews and Meta-Analyses (PRISMA 2020) guidelines (Supplementary File S1 [10]). An electronic literature search was performed using the PubMed/MEDLINE, SCOPUS, Web of Science, and Cochrane Library databases, with searches conducted up to 16 June 2026 The review protocol was prospectively submitted for registration in PROSPERO (CRD420261508554).

The search strategy included studies published from database inception through June 2026. A combination of Medical Subject Headings (MeSH) terms and free-text keywords related to articaine infiltration anesthesia in primary molars was used. The following search strategy was applied: (“articaine”) AND (“buccal infiltration” OR “infiltration anesthesia”) AND (“primary molars” OR “deciduous molars” OR “primary teeth”) AND (“children” OR “pediatric dentistry”) (Supplementary File S2). 

Additionally, manual screening of the reference lists of the included studies was performed to identify potentially relevant publications not retrieved through the electronic search.

### 2.2. Study Selection

The research question was formulated according to the PICO framework:

- Population (P): Pediatric patients undergoing dental procedures involving primary mandibular molars.
- Intervention (I): Buccal infiltration with 4% articaine.
- Comparison (C): Inferior alveolar nerve block with 2% lidocaine or buccal infiltration with lidocaine.
- Outcome (O): Anesthetic success, pain during injection and dental treatment, need for supplemental anesthesia, behavioral response, and postoperative adverse effects.

Two reviewers independently screened the titles and abstracts of all retrieved records. Potentially eligible studies were subsequently assessed independently by the same two reviewers through full-text evaluation according to the predefined inclusion and exclusion criteria. Disagreements between the reviewers were resolved through discussion and consensus. When consensus could not be reached, a third reviewer was consulted.

The inclusion criteria were:

1. randomized clinical trials;
2. split-mouth or crossover randomized clinical studies;
3. studies involving pediatric patients;
4. studies evaluating primary mandibular molars;
5. studies assessing buccal infiltration with 4% articaine;
6. studies reporting at least one clinically relevant anesthetic outcome, including anesthetic success, pain, need for supplemental anesthesia, behavioral response, or postoperative adverse effects.

The exclusion criteria were:

1. studies involving permanent teeth only;
2. studies conducted in adults;
3. review articles and meta-analyses;
4. case reports;
5. animal or in vitro studies.

### 2.3. Data Extraction and Analysis

Data extraction was performed independently by two reviewers using a standardized data-extraction form. The extracted information included study characteristics, sample size, participant characteristics, anesthetic interventions and comparators, dental procedures, outcome measures, and main clinical findings. Any discrepancies between the reviewers were resolved through discussion and consensus, with consultation of a third reviewer when necessary.

The primary outcomes included anesthetic success and pain intensity during treatment. Secondary outcomes included the need for supplemental anesthesia, postoperative soft tissue trauma, behavioral response during treatment, and adverse events. Pain assessment methods included the Wong-Baker Faces Pain Rating Scale (WBFRS), Visual Analogue Scale (VAS), Face, Legs, Activity, Cry, Consolability Scale (FLACC), Modified Behavioral Pain Scale (MBPS), and Faces Pain Scale—Revised (FPS-R). Behavioral response was assessed using the Frankl Behavior Rating Scale (FBRS).

A qualitative synthesis of the included studies was conducted. Although four of the five included trials evaluated 4% articaine buccal infiltration against 2% lidocaine using mandibular anesthesia techniques, quantitative pooling was considered inappropriate because of important differences in clinical procedures, outcome definitions, pain assessment instruments, and reporting formats. In particular, pain outcomes were assessed using different pediatric pain and behavioral scales, while anesthetic success was defined and reported differently across studies. Furthermore, the numerical data and measures of variance required for calculation of a common effect estimate were not consistently reported across the included studies. Therefore, a meta-analysis would have required substantial assumptions or transformation of incompletely reported data, which could have introduced additional uncertainty and reduced the reliability of the pooled estimates. The findings were consequently synthesized narratively.

### 2.4. Risk of Bias in Individual Studies

The risk of bias of the included randomized controlled trials was assessed independently by two reviewers using the Cochrane Risk of Bias 2 (RoB 2) tool (version 2). The assessment was performed independently by both reviewers across the five RoB 2 domains, with judgments categorized as “low risk,” “some concerns,” or “high risk.” Any disagreements between the reviewers were resolved through discussion and consensus; when necessary, a third reviewer was consulted. The appropriate RoB 2 guidance was applied according to the study design, including parallel-group, split-mouth, and crossover trials, where applicable.

## 3. Results

### 3.1. Search

The literature search identified five randomized controlled clinical trials that fulfilled the predefined eligibility criteria. Following title and abstract screening, potentially eligible reports were assessed by full-text evaluation. Of the 23 reports assessed for eligibility, 18 were excluded for the reasons specified in the PRISMA flow diagram, and five studies were included in the qualitative synthesis. No additional eligible studies were identified through manual searching of reference lists. The study selection process is illustrated in Figure 1.

Overall, five randomized controlled trials [11,12,13,14,15] involving 327 pediatric patients aged between 5 and 10 years were included. Four studies compared 4% articaine administered by buccal infiltration with 2% lidocaine delivered by IANB, whereas one study compared buccal infiltration of both anesthetic agents.

### 3.2. Characteristics of the Included Studies

Although all studies addressed the same clinical question, several methodological differences were observed. Sample sizes ranged from 28 to 112 participants, and studies were conducted in five different countries (Table 1). Three investigations adopted a parallel-group randomized design, whereas one employed a split-mouth design and one used a crossover methodology. Although the eligibility criteria permitted studies involving dental procedures on primary mandibular molars, four of the five included studies evaluated anesthesia during extraction of primary mandibular molars, while one study also included pulpotomy procedures. Therefore, the qualitative synthesis primarily reflects the clinical use of buccal articaine infiltration in procedures involving primary mandibular molars.

Clinical protocols were generally comparable. All studies used epinephrine-containing anesthetic solutions, while pain perception and behavioral response were assessed using validated pediatric pain scales. The most frequently reported outcomes included intraoperative pain, anesthetic success, behavioral response, need for supplemental anesthesia, and postoperative complications.

### 3.3. Outcome of the Included Studies

Overall, all five studies demonstrated that buccal infiltration using 4% articaine achieved anesthetic efficacy comparable to conventional 2% lidocaine. Four studies directly compared articaine infiltration with IANB and consistently reported no statistically significant differences in pain experienced during extraction or overall anesthetic success. The remaining study, which compared buccal infiltration of both anesthetic agents, similarly demonstrated equivalent clinical effectiveness (Table 2).

Although the overall anesthetic efficacy was comparable, minor differences were observed regarding discomfort during anesthetic administration. Three studies reported no significant differences between anesthetic techniques, whereas Jain et al. [13] observed improved behavioral responses during injection with articaine, and Massignan et al. [14] reported slightly greater pain during deposition of the articaine solution. Importantly, these findings did not influence treatment success during extraction. 

No serious adverse events were reported. Soft tissue trauma, particularly accidental lip biting, occurred more frequently following IANB than buccal infiltration, representing the most consistent clinical advantage associated with the infiltration technique. Other postoperative complications, including edema and transient discomfort, were uncommon and resolved without intervention (Table 3). 

Despite variations in study design and outcome assessment methods, a remarkably consistent pattern emerged. None of the included randomized controlled trials demonstrated inferior anesthetic efficacy of buccal infiltration with articaine during extraction of primary molars. Four studies ultimately favored articaine as a clinically acceptable alternative to IANB, while one study reported equivalent outcomes without demonstrating superiority of either anesthetic approach (Table 4).

### 3.4. The Risk of Bias in the Individual Studies

All included studies were randomized controlled trials. Four studies adequately described random sequence generation and allocation concealment, while one study provided limited methodological details regarding allocation procedures. Missing outcome data were minimal across all investigations, and selective reporting was considered unlikely. Outcome assessment was generally performed using validated pain and behavioral scales, reducing the risk of measurement bias (Figure 2).

Performance bias represented the principal methodological limitation because operator blinding was not feasible in several studies due to differences in anesthetic techniques. Nevertheless, this limitation is inherent to clinical anesthesia trials and was partially minimized through blinded outcome assessment in most investigations. Overall, the included studies were considered to have low risk or some concerns regarding the risk of bias, supporting the reliability of the synthesized evidence.

## 4. Discussion

This systematic review aimed to evaluate the clinical efficacy of buccal infiltration with 4% articaine during dental procedures involving primary molars in pediatric patients. Based on the currently available evidence, buccal infiltration with articaine demonstrated favorable clinical outcomes, with anesthetic efficacy comparable to that of conventional inferior alveolar nerve block (IANB), while providing effective pain control and favorable patient comfort. These findings suggest that buccal infiltration with articaine may represent a minimally invasive alternative to conventional IANB for selected dental procedures in pediatric dentistry.

One of the most important findings of this review was the consistently high anesthetic success reported across the included studies. Buccal infiltration with articaine provided clinically acceptable anesthesia during procedures involving primary mandibular molars, predominantly extractions. Traditionally, mandibular procedures have been considered to require IANB because of the thickness of the cortical bone and the potentially limited diffusion of local anesthetic solutions. However, the findings of this review suggest that these considerations may not fully apply to primary mandibular molars when articaine is used for buccal infiltration.

The comparable anesthetic efficacy of articaine and lidocaine may be explained, at least in part, by the pharmacological properties of articaine. Unlike other amide local anesthetics, articaine contains a thiophene ring, which increases lipid solubility and may facilitate tissue penetration. This characteristic may promote diffusion through soft tissues and bone, allowing the anesthetic solution to reach the apical region of the tooth more effectively. In addition, articaine exhibits greater protein binding than lidocaine, which may contribute to a longer duration of anesthesia and favorable clinical performance [7]. These pharmacological characteristics may contribute to the comparable outcomes observed with articaine infiltration and conventional mandibular nerve block techniques.

The anatomical characteristics of the primary dentition may further contribute to the effectiveness of infiltration anesthesia. The cortical bone surrounding primary molars is generally thinner and less dense than that surrounding permanent teeth. In addition, primary teeth have shorter roots and are surrounded by a relatively greater proportion of cancellous bone [8]. These anatomical features may facilitate diffusion of the anesthetic solution toward the neural structures supplying the tooth. Consequently, adequate pulpal anesthesia may be achieved with infiltration alone in selected clinical situations, without the need for a deeper nerve block technique.

Pain control is one of the most important determinants of successful dental treatment in children. Several studies included in this review reported lower pain scores with articaine infiltration compared with conventional mandibular nerve block. This finding is particularly relevant because the administration of local anesthesia itself is often one of the more stressful aspects of dental treatment for pediatric patients. The less invasive nature of buccal infiltration, reduced needle penetration, and less extensive tissue manipulation may contribute to a more comfortable experience for children.

Improved patient cooperation was another finding reported in several of the included studies. Children receiving buccal infiltration often demonstrated favorable behavioral responses during treatment. This observation has important clinical implications because behavior management remains a fundamental component of pediatric dentistry. A less traumatic anesthetic experience may contribute to reduced dental fear and facilitate subsequent dental visits. Therefore, when clinically appropriate, achieving effective anesthesia using a simpler injection technique may provide immediate benefits and potentially contribute to more favorable future dental experiences.

A particularly relevant finding was the lower incidence of postoperative soft tissue injuries associated with infiltration anesthesia. IANB can result in prolonged numbness of the lower lip, cheek, and tongue [16]. Young children may not fully understand the consequences of soft tissue anesthesia and may inadvertently bite anesthetized tissues, resulting in ulceration, swelling, pain, and parental concern. Several studies included in this review reported a lower frequency of lip and cheek biting in children treated with buccal infiltration. This finding may represent an important clinical advantage of infiltration anesthesia in routine pediatric dental practice.

The findings of this review are also consistent with contemporary concepts of minimally invasive pediatric dentistry [17]. Modern pediatric dentistry increasingly emphasizes minimizing discomfort while maintaining effective clinical outcomes. Minimally invasive approaches include interventions aimed at reducing both psychological and physical trauma during treatment. In this context, using a simpler infiltration technique instead of IANB, when clinically appropriate, is consistent with the principles of contemporary patient-centered dental care.

Although the overall findings were favorable, some variability among the included studies was observed. Differences existed in the types of procedures performed, outcome assessment methods, and comparator interventions. Most studies evaluated dental extractions, while one study also included pulpotomy procedures. Anesthetic requirements may differ between these procedures, particularly because pulpotomy involves direct manipulation of pulpal tissue and therefore requires adequate pulpal anesthesia. Therefore, caution should be exercised when generalizing the findings beyond the clinical procedures evaluated in the included studies.

An important methodological consideration is that a meta-analysis was not performed despite the clinical similarity of several included trials. Four studies compared 4% articaine buccal infiltration with 2% lidocaine administered using conventional mandibular anesthesia techniques. However, the available data were not sufficiently harmonized to support reliable quantitative synthesis. Differences in clinical procedures, definitions of anesthetic success, pain measurement instruments, and reporting of variance estimates limited the comparability of the reported outcomes. Consequently, pooling the results would have required assumptions or statistical transformations that could have introduced additional uncertainty. A narrative synthesis was therefore considered the most appropriate approach for the available evidence. Future studies using standardized outcome definitions and consistent reporting of effect estimates and measures of variance would facilitate more robust meta-analytic evaluation.

The need for supplemental anesthesia remains an important clinical consideration. Although most studies reported high success rates following a single buccal infiltration, additional anesthesia was occasionally required, particularly during more invasive procedures. This finding suggests that articaine infiltration cannot completely eliminate the need for supplemental anesthetic techniques in all clinical situations. Nevertheless, even when supplemental anesthesia is required, initiating treatment with a minimally invasive infiltration approach may offer practical advantages compared with routine administration of IANB.

The risk-of-bias assessment indicated generally acceptable methodological quality among the included studies. Most investigations employed randomization and clearly described their intervention protocols. However, some studies had relatively small sample sizes, potentially limiting statistical power. In addition, blinding procedures were not always described in sufficient detail, and the use of different pain assessment scales may have contributed to variability in the reported outcomes. These limitations should be considered when interpreting the findings.

Compared with systematic reviews published in 2021, the present review provides an updated and more focused synthesis of the evidence regarding buccal infiltration with articaine for anesthesia of primary mandibular molars in children [8,9]. The present review incorporated more recent evidence identified through an updated literature search and applied predefined eligibility criteria focused specifically on the pediatric population, primary mandibular molars, and the comparison of buccal infiltration with articaine and conventional inferior alveolar nerve block. In addition, the current review provides a detailed assessment of the methodological quality of the included randomized controlled trials using the RoB 2 tool and presents the available outcomes according to specific clinical procedures and outcome measures. Despite the updated evidence base, the small number of eligible studies and their clinical and methodological heterogeneity remain important limitations and preclude firm conclusions regarding the superiority of articaine.

From a clinical perspective, these findings may have relevant implications for pediatric dental practice. Pediatric dentists seek to balance effective pain control with minimizing anxiety and treatment-related complications. Buccal infiltration with articaine may help achieve these objectives in selected clinical situations. The technique is relatively simple to administer and was generally associated with favorable behavioral responses and fewer postoperative soft tissue complications while maintaining comparable anesthetic efficacy to conventional IANB.

Future research should focus on multicenter randomized clinical trials with larger sample sizes and standardized outcome assessment methods. Further investigations are also needed to evaluate the effectiveness of articaine infiltration during specific procedures, such as pulpotomies, pulpectomies, stainless steel crown placement, and extraction of severely destroyed primary molars. Incorporating patient-reported outcomes, parental satisfaction measures, and cost-effectiveness analyses may provide a more comprehensive understanding of the overall clinical value of this technique.

## 5. Conclusions

The available evidence suggests that buccal infiltration with 4% articaine may provide comparable anesthetic efficacy to that of conventional lidocaine inferior alveolar nerve block in primary mandibular molars undergoing extraction or pulpotomy. However, the current evidence does not support the conclusion that articaine is superior to lidocaine. Given the limited number of eligible studies and their clinical and methodological heterogeneity, the findings should be interpreted with caution. Buccal infiltration with 4% articaine may represent a useful and less invasive alternative to inferior alveolar nerve block for selected pediatric extraction and pulpotomy procedures. Further well-designed randomized controlled trials using standardized outcome measures are needed to establish its comparative effectiveness and define the clinical situations in which this approach may provide the greatest benefit.

## Figures and Tables

**Figure 1:**
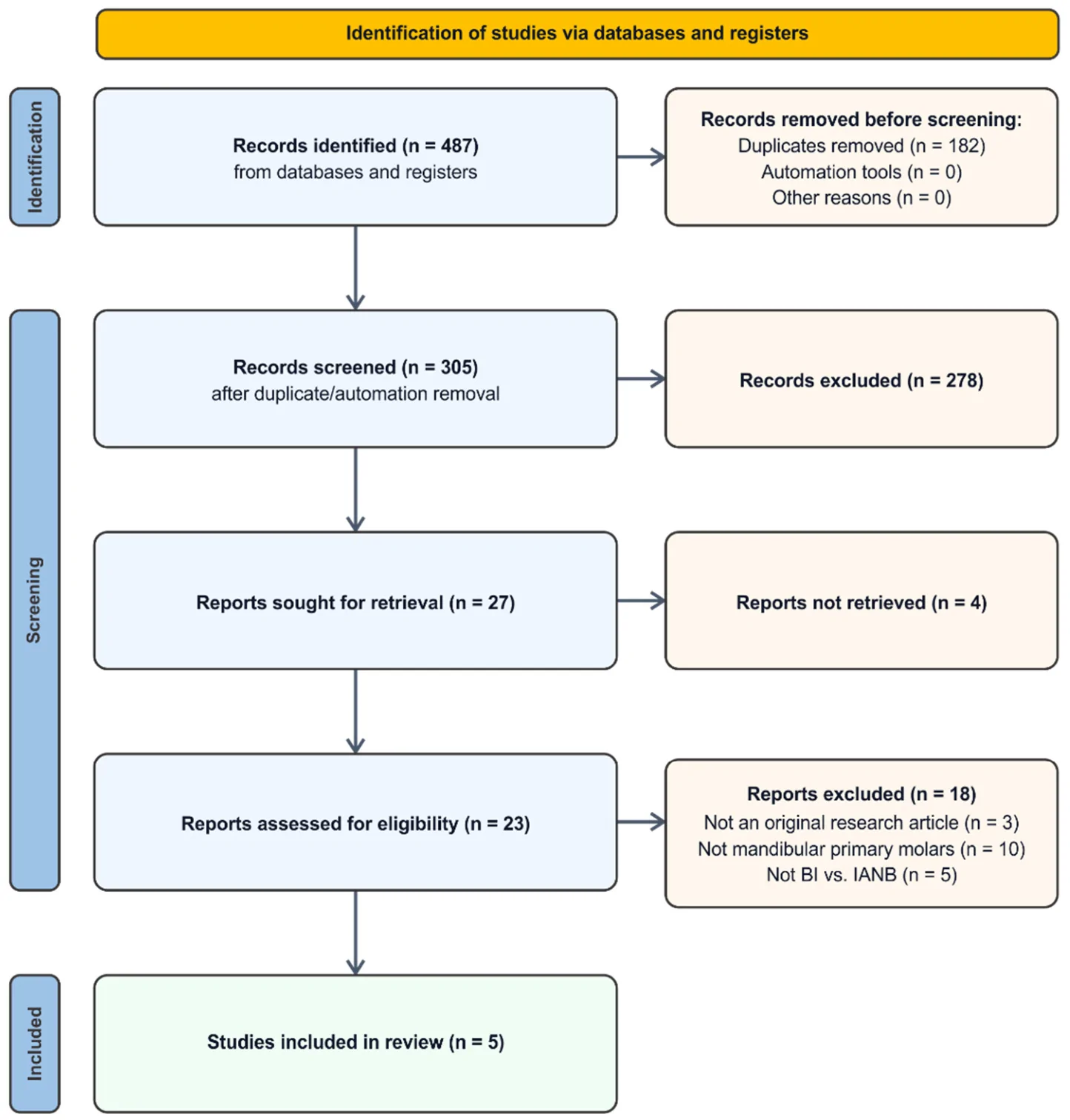
PRISMA 2020 flow diagram of study selection.

**Figure 2:**
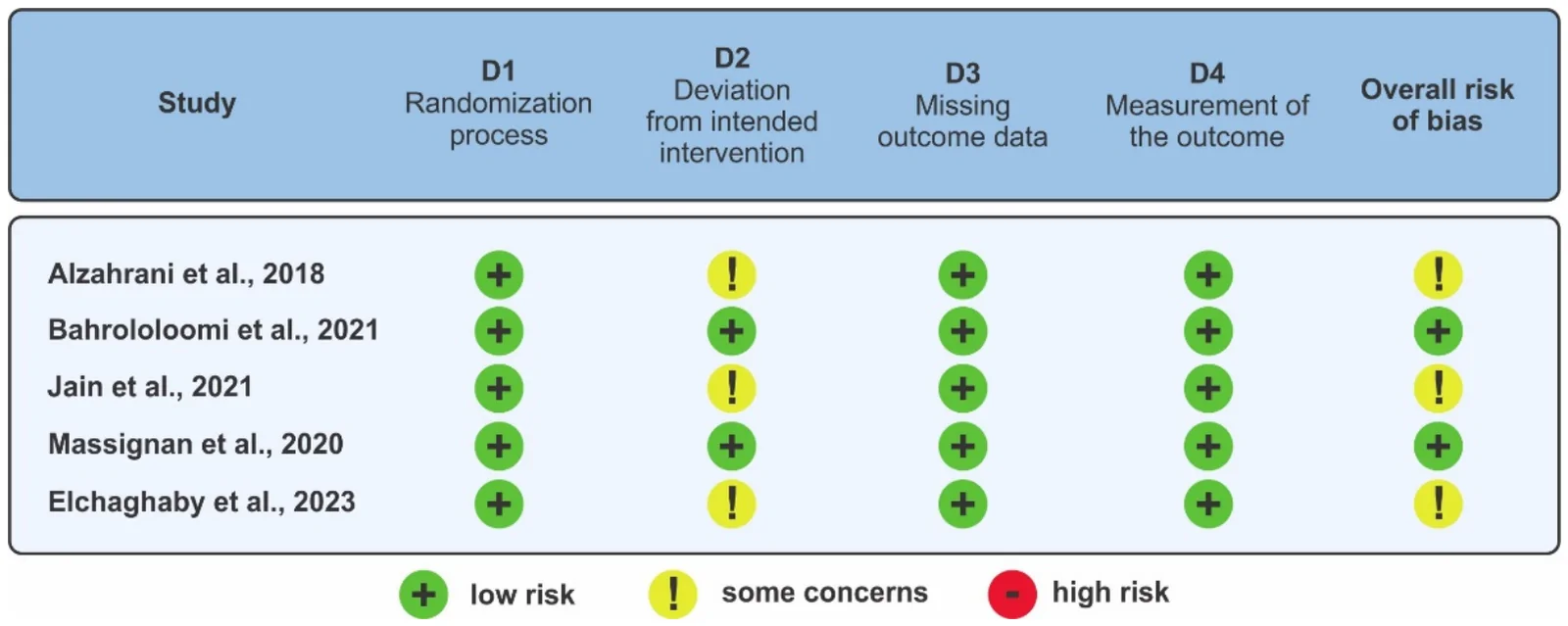
Quality assessment of the included RCT according to the Cochrane Risk of Bias 2 tool. [11,12,13,14,15].

**Table 1:** Characteristics of the included studies.

Study	Country	Study Design	Sample Size	Age (Years)	Intervention	Comparator	Procedure	Outcomes
Alzahrani et al., 2018 [11]	UK	Parallel RCT	98	5–9	BI (4% Articaine)	IANB (2% Lidocaine)	Extraction and Pulpotomy	WBFRS, VAS, FBRS
Bahrololoomi et al., 2021 [12]	Iran	Crossover RCT	28	6–9	BI (4% Articaine)	IANB (2% Lidocaine)	Bilateral extraction	FLACC, WBFRS
Jain et al., 2021 [13]	India	Split-mouth RCT	46	5–10	BI (4% Articaine)	IANB (2% Lidocaine)	Bilateral extraction	WBFRS, MBPS, FBRS
Massignan et al., 2020 [14]	Brazil	Triple-blind RCT	43	6–10	BI (4% Articaine)	IANB (2% Lidocaine)	Extraction	FPS-R
Elchaghaby et al., 2023 [15]	Egypt	Parallel RCT	112	5–8	BI (4% Articaine)	IANB (2% Lidocaine)	Extraction	FLACC, WBFRS

**Table 2:** Comparative clinical outcomes.

Study	Injection Pain	Extraction Pain	Additional Anesthesia	Main Conclusion
Alzahrani et al., 2018 [11]	W-BFRS: equal success (63.3%). VAS: 73.5% BI vs. 79.6% IANB; nonequivalence.	W-BFRS: 70.8% BI vs. 67.3% IANB; nonequivalence. VAS: 81.6% BI vs. 79.6% IANB; equivalent.	Similar 1 IANB/3 BI	Equivalent efficacy
Bahrololoomi et al., 2021 [12]	No statistically significant difference between groups. FLACC: lidocaine 0.89 vs. articaine 1.36. WBFPS: no significant between-group difference.	No statistically significant difference between groups	Not reported	Equivalent efficacy
Jain et al., 2021 [13]	Lower pain with articaine; statistically significant for crying (MBPS): 1.30 vs. 1.76 with lidocaine (*p* = 0.024). W-BFRS showed no significant difference.	No significant difference	No significant difference	Comparable efficacy; favorable for BI
Massignan et al., 2020 [14]	Higher pain with BI (articaine): mean increase of 2.43 FPS-R units vs. lidocaine (95% CI 0.28–4.57)	No significant difference: pain-free in 42.9% with BI vs. 31.8% with IANB	Not reported	Comparable extraction pain; IANB favored for injection pain
Elchaghaby et al., 2023 [15]	No significant difference; W-BFPRS scores comparable	No significant difference; W-BFPRS scores comparable	Not reported	Comparable efficacy; faster onset and shorter duration with BI

**Table 3:** Postoperative complications.

Study	Lip Biting/Soft Tissue Injuries	Nausea in 24 h
Alzahrani et al., 2018 [11]	1 IANB/1 BI	Not reported
Bahrololoomi et al., 2021 [12]	Not reported	Not reported
Jain et al., 2021 [13]	4 IANB/2 BI	Not reported
Massignan et al., 2020 [14]	Not reported	2 IANB/3 BI
Elchaghaby et al., 2023 [15]	Significantly more frequent in the IANB	Not reported

**Table 4:** Overall direction of treatment effects.

Outcome	Studies Favoring Articaine	Equivalent	Favoring Lidocaine
Injection pain	1	3	1
Pain during extraction	0	5	0
Anesthetic success	0	5	0
Need for supplemental anesthesia	0	5	0
Postoperative complications	4	1	0

## Data Availability

No new data were created or analyzed in this study. Data sharing is not applicable to this article.

## Supplementary Materials

The following supporting information can be downloaded at: https://www.mdpi.com/article/10.3390/dj14100620/s1, File S1 PRISMA 2020 Checklist; File S2: Complete search strategies used for the electronic databases.

## Abbreviations

The following abbreviations are used in this manuscript:

IANB	Inferior Alveolar Nerve Block
BI	Buccal Infiltration of Articaine
RCT	Randomized Controlled Trials
WBFRS	Wong–Baker Faces Pain Rating Scale
VAS	Visual Analogue Scale
FBRS	Frankl Behavior Rating Scale
FLACC	Face, Legs, Activity, Cry, Consolability Scale
MBPS	Modified Behavioral Pain Scale
FPS-R	Faces Pain Scale—Revised
