# Supplementary material for: Buccal Articaine Infiltration as a Minimally Invasive Technique for Mandibular Primary Molars: A Systematic Review
Source: Dent J (Basel). 2026 Sep 26;14(10):620. doi: 10.3390/dj14100620 (PMC13702935; doi:10.3390/dj14100620)
Supplement: Supplementary file 1 [file dentistry-14-00620-s001.zip › Supplementary File S2.pdf]

Supplementary File S2

Database Search Strategies

Buccal Articaine Infiltration as a Minimally Invasive Technique for Mandibular Primary Molars: A Systematic Review

The literature search was conducted in PubMed/MEDLINE, Scopus, Web of Science Core Collection, and the Cochrane Library. Searches covered the period from database inception through June 2026. Database-specific search strategies were adapted to the indexing systems and search syntax of each database while retaining the same core concepts: articaine, buccal/infiltration anesthesia, primary molars or primary teeth, and children/pediatric dentistry.

S1. PubMed/MEDLINE

The PubMed/MEDLINE strategy combined Medical Subject Headings (MeSH) and free-text keywords:

```
(“articaine”) AND (“buccal infiltration” OR “infiltration anesthesia”) AND (“primary
molars” OR “deciduous molars” OR “primary teeth”) AND (“children” OR “pediatric dentistry”)
```

S2. Scopus

```
TITLE-ABS-KEY (articaine) AND TITLE-ABS-KEY ("buccal infiltration" OR "infiltration
anesthesia" OR "infiltration anaesthesia") AND TITLE-ABS-KEY ("primary molars" OR
"deciduous molars" OR "primary teeth") AND TITLE-ABS-KEY (children OR pediatric OR
paediatric OR "pediatric dentistry" OR "paediatric dentistry")
```

S3. Web of Science Core Collection

```
TS=(articaine) AND TS=("buccal infiltration" OR "infiltration anesthesia" OR "infiltration
anaesthesia") AND TS=("primary molars" OR "deciduous molars" OR "primary teeth") AND
TS=(children OR pediatric OR paediatric OR "pediatric dentistry" OR "paediatric dentistry")
```

S4. Cochrane Library

```
articaine AND ("buccal infiltration" OR "infiltration anesthesia" OR "infiltration
anaesthesia") AND ("primary molars" OR "deciduous molars" OR "primary teeth") AND (children
OR pediatric OR paediatric OR "pediatric dentistry" OR "paediatric dentistry")
```

S5. Search Limits

No language restriction was applied during the database search. No publication type restriction was applied at the initial search stage. Records were subsequently screened according to the predefined eligibility criteria of the systematic review.

S6. Search Date

All database searches were conducted from database inception through June 2026.

Table S1. Database-specific search syntax

Database	Search field / syntax
PubMed/MEDLINE	MeSH terms + free-text keywords
Scopus	TITLE-ABS-KEY
Web of Science Core Collection	TS (Topic)
Cochrane Library	Keyword search
